# Supplementary material for: Make-or-break prime editing for genome engineering in Streptococcus pneumoniae
Source: Nat Commun. 2025 Apr 23;16:3796. doi: 10.1038/s41467-025-59068-8 (PMC12015366; doi:10.1038/s41467-025-59068-8)
Supplement: Supplementary file 2 — Description of Additional Supplementary Files [file 41467_2025_59068_MOESM2_ESM.pdf]

Description of Additional Supplementary Files

File Name: Supplementary Data 1
Description: Oligonucleotides included in the ssDNA pegRNA pool ordered from Twist Bioscience.

File Name: Supplementary Data 2
Description: Strains and plasmids used in this study.

File Name: Supplementary Data 3
Description: Primers used in this study.

File Name: Supplementary Data 4
Description: Results of analysis of *luc* sequencing using DESeq2. For each type of modification encoded in the pegRNA pool (see Supplementary Data 1), a differential abundance analysis was performed using DESeq2. For each modification, the resulting statistics are noted when available. Testing was performed using the Wald test, and an absolute log2FoldChange > 1, and a p-value of 0.05. Statistical testing was compensated for multiple testing according to standard settings of DESeq2.For each type of modification, the raw counts of the number of reads observed having that mutation, the DESeq2 normalized count, fraction, mean, and SD are indicated.

File Name: Supplementary Data 5
Description: Results of analysis of pegRNA sequencing using DESeq2. For each type of modification encoded in the pegRNA pool (see Supplementary Data 1), a differential abundance analysis was performed using DESeq2. For each modification, the resulting statistics are noted when available. Testing was performed using the Wald test, and an absolute log2FoldChange > 1, and a p-value of 0.05. Statistical testing was compensated for multiple testing according to standard settings of DESeq2. For each type of modification, the raw counts of the number of reads observed having that mutation, the DESeq2 normalized count, fraction, mean, and SD are indicated.
