# Supplementary material for: Make-or-break prime editing for genome engineering in Streptococcus pneumoniae
Source: Nat Commun. 2025 Apr 23;16:3796. doi: 10.1038/s41467-025-59068-8 (PMC12015366; doi:10.1038/s41467-025-59068-8)
Supplement: Supplementary file 8 — Reporting Summary [file 41467_2025_59068_MOESM8_ESM.pdf]

nature portfolio

Corresponding author(s): Jan-Willem Veening

Last updated by author(s): Mar 17, 2025



# Reporting Summary

Nature Portfolio wishes to improve the reproducibility of the work that we publish. This form provides structure for consistency and transparency in reporting. For further information on Nature Portfolio policies, see our Editorial Policies and the Editorial Policy Checklist.

## Statistics

For all statistical analyses, confirm that the following items are present in the figure legend, table legend, main text, or Methods section.

| n/a | Confirmed | |
|---|---|---|
| ☐ | ☒ | The exact sample size (*n*) for each experimental group/condition, given as a discrete number and unit of measurement |
| ☐ | ☒ | A statement on whether measurements were taken from distinct samples or whether the same sample was measured repeatedly |
| ☐ | ☒ | The statistical test(s) used AND whether they are one- or two-sided<br>*Only common tests should be described solely by name; describe more complex techniques in the Methods section.* |
| ☒ | ☐ | A description of all covariates tested |
| ☒ | ☐ | A description of any assumptions or corrections, such as tests of normality and adjustment for multiple comparisons |
| ☐ | ☒ | A full description of the statistical parameters including central tendency (e.g. means) or other basic estimates (e.g. regression coefficient) AND variation (e.g. standard deviation) or associated estimates of uncertainty (e.g. confidence intervals) |
| ☐ | ☒ | For null hypothesis testing, the test statistic (e.g. *F*, *t*, *r*) with confidence intervals, effect sizes, degrees of freedom and *P* value noted<br>*Give P values as exact values whenever suitable.* |
| ☒ | ☐ | For Bayesian analysis, information on the choice of priors and Markov chain Monte Carlo settings |
| ☒ | ☐ | For hierarchical and complex designs, identification of the appropriate level for tests and full reporting of outcomes |
| ☒ | ☐ | Estimates of effect sizes (e.g. Cohen's *d*, Pearson's *r*), indicating how they were calculated |

*Our web collection on statistics for biologists contains articles on many of the points above.*

## Software and code

Policy information about availability of computer code

| | |
|---|---|
| Data collection | No custom software or code was used to collect the data |
| Data analysis | Sequencing reads were analyzed using 2FAST2Q (v2.5.3). Differential abundance analysis was performed in Rstudio using DESeq2 (v1.40.2). Microscopy images were analysed using Fiji (v1.54g), deconvolution of signals was performed using Huygens (v17.10.0p4), and demograph plots were formed using MicrobeJ (v5.13h). |

For manuscripts utilizing custom algorithms or software that are central to the research but not yet described in published literature, software must be made available to editors and reviewers. We strongly encourage code deposition in a community repository (e.g. GitHub). See the Nature Portfolio guidelines for submitting code & software for further information.

## Data

Policy information about availability of data

All manuscripts must include a data availability statement. This statement should provide the following information, where applicable:

- Accession codes, unique identifiers, or web links for publicly available datasets
- A description of any restrictions on data availability
- For clinical datasets or third party data, please ensure that the statement adheres to our policy

The sequencing data from sequencing reactions of the luciferase gene and the pegRNAs, and the PacBio sequencing data generated in this study have been

deposited the Sequence Read Archive under BioProject accession code PRJNA1062582 [https://www.ncbi.nlm.nih.gov/bioproject/?term=PRJNA1062582]. Source data are provided with this paper.

## Research involving human participants, their data, or biological material

Policy information about studies with human participants or human data. See also policy information about sex, gender (identity/presentation), and sexual orientation and race, ethnicity and racism.

| | |
|---|---|
| Reporting on sex and gender | As no human participants were involved in this study, no sex and/or gender information is reported. |
| Reporting on race, ethnicity, or other socially relevant groupings | As no human participants were involved in this study, no race, ethnicity, or other socially relevant groupings are reported. |
| Population characteristics | As no human participants were involved in this study, there are no population characteristics |
| Recruitment | As no human participants were involved in this study, no recruitment was performed |
| Ethics oversight | As no human participants were involved in this study, no ethics oversight was necessary for this topic. |

Note that full information on the approval of the study protocol must also be provided in the manuscript.

# Field-specific reporting

Please select the one below that is the best fit for your research. If you are not sure, read the appropriate sections before making your selection.

☒ Life sciences ☐ Behavioural & social sciences ☐ Ecological, evolutionary & environmental sciences

For a reference copy of the document with all sections, see nature.com/documents/nr-reporting-summary-flat.pdf

# Life sciences study design

All studies must disclose on these points even when the disclosure is negative.

| | |
|---|---|
| Sample size | No statistical method was used to predetermine sample size. Sample size for luciferase activity experiments was determined by the physical limitation of the 96 well plates used in the experimental setup. For the pegRNA and luciferase sequencing experiments, four separate biological screenings with the created library were performed. |
| Data exclusions | No data were excluded from the analyses. |
| Replication | Reproducibility of the experimental findings was strengthend by performing repeated biological replicates. |
| Randomization | The experiments were not randomized. The unrandomized nature was controlled for using unbiased methods and resources during experimental setups and analyses. |
| Blinding | The Investigators were not blinded to allocation during experiments and outcome assessment. Recording of experimental outcomes and data analysis required unblinded workflows. |

# Reporting for specific materials, systems and methods

We require information from authors about some types of materials, experimental systems and methods used in many studies. Here, indicate whether each material, system or method listed is relevant to your study. If you are not sure if a list item applies to your research, read the appropriate section before selecting a response.

## Materials & experimental systems

| n/a | Involved in the study | |
|---|---|---|
| ☒ | ☐ | Antibodies |
| ☒ | ☐ | Eukaryotic cell lines |
| ☒ | ☐ | Palaeontology and archaeology |
| ☒ | ☐ | Animals and other organisms |
| ☒ | ☐ | Clinical data |
| ☒ | ☐ | Dual use research of concern |
| ☒ | ☐ | Plants |

## Methods

| n/a | Involved in the study | |
|---|---|---|
| ☒ | ☐ | ChIP-seq |
| ☒ | ☐ | Flow cytometry |
| ☒ | ☐ | MRI-based neuroimaging |

## Plants

| | |
|---|---|
| Seed stocks | Not applicable |
| Novel plant genotypes | Not applicable |
| Authentication | Not applicable |
